# Dr. John N. Frink

**Published:** 1844-12

**Authors:** 


*Obituary.*—Died, at his residence in Portland, Maine, of pulmonary consumption, JOHN N. FRINK, M. D., Fellow of the American Society of Dental Surgeons, at the age of 35 years. Dr. Frink was for many years a practitioner of Dental Surgery in Portland, and was highly esteemed for talent, skill and integrity in his profession, Our personal acquaintance with the deceased was but slight, but it was sufficient to leave on our memory a very favorable impression of his character. M.

